# Epigenetics & chromatin: interactions and processes

**DOI:** 10.1186/1756-8935-6-2

**Published:** 2013-02-27

**Authors:** Steven Henikoff, Frank Grosveld

**Affiliations:** 1 Howard Hughes Medical Institute and Basic Sciences Division, Fred Hutchinson Cancer Research Center, 1100 Fairview Avenue North, WA, 98109-1024, Seattle, USA; 2 Department of Cell Biology, Erasmus Medical Center, Dr. Molewaterplein 50, Rotterdam, The Netherlands

## Abstract

On 11 to 13 March 2013, BioMed Central will be hosting its inaugural conference, Epigenetics & Chromatin: Interactions and Processes, at Harvard Medical School, Cambridge, MA, USA. *Epigenetics & Chromatin* has now launched a special article series based on the general themes of the conference.

## 

Over the past two decades we have witnessed the merging of two very different fields of study, epigenetics and chromatin biochemistry. Epigenetic phenomena were first described in the 1920s with the discovery of genetically stable mutant alleles in *Drosophila* and maize that displayed variegated clonal expression patterns. The field of chromatin biochemistry began in earnest decades later, revolutionized in the early 1970s with the discovery that chromosomes are organized into nucleosomes followed by the realization that different parts of the genome interact with each other. Both epigenetics and chromatin biochemistry were successfully applied to the study of mechanisms responsible for gene regulation and developmental programming in several model organisms and in humans. By the end of the 20th century, it had become clear that the effects on gene expression observed in epigenetic phenomena and during development could only be fully understood in light of chromatin-based mechanisms. While traditional genetic and biochemical approaches to studying genetic regulatory mechanisms continue, the trend has been to apply epigenetic and chromatin methodologies in combination, especially taking advantage of innovations in proteomics, genomics, microscopy and other technologies, combined with the impressive progress in informatics.

Mergers do not come easily, and experts in one or the other discipline have much to learn from one another, both in terms of subject matter and technology. Epigenetics, driven in part by studies of gene and chromosome function, has documented a plethora of nuclear phenomena that need to be explained, including developmental decision-making, heritable gene silencing, heterochromatin formation, dosage compensation and the development of diseases such as cancer. Chromatin biochemistry, driven in part by genomic technologies, has identified proteins and other molecules that self-organize within the nucleus to form dynamic chromatin configurations that ultimately are responsible for chromosomal processes, such as replication, transcription, mitosis, repair and recombination. The daunting biological complexity of the even simple model systems and the many different experimental strategies that have been developed to deal with this complexity motivate a broadly based meeting centered around basic processes at the intersection of epigenetics and chromatin.

The conference [1] organizers have identified 10 broad topics with a strong emphasis on processes and interactions. This emphasis is justified by recent exciting progress in this rapidly changing field. For example, after years of describing static chromatin modifications, we are beginning to gain a glimpse into the dynamics of nucleosome assembly and modification, the importance of chromatin remodelers, histone chaperones and transcriptional elongation complexes, and clarification of their roles in development and disease. Other topics are intended to span the range of interests of practitioners in the field. After decades of thinking that methylation is the only covalent modification of DNA, we have been surprised in just the past few years by the discovery of a methylcytosine oxidation pathway with important implications for the stability of this epigenetic mark. Similarly, although much is now known about how small RNAs act on cytoplasmic and nascent RNAs, we have as yet only scratched the surface in understanding how long non-coding RNAs act to mediate epigenetic processes, and it remains to be seen how prevalent they are in regulating gene expression. The molecular basis for epigenetic memory during development remains as yet an unsolved problem, but enough is now known about the players involved that there is reason to expect breakthroughs soon. Finally, the large-scale organization of the nucleus has undergone a dramatic increase in resolution thanks to technological progress in both genomics and microscopy.

Based on the epigenetic interactions and processes highlighted in the sessions, we have decided to launch a new thematic series as a permanent record of the invaluable research presented at the conference. The series will feature short Reviews, Commentaries, Opinion pieces and Research written by the speakers, and will be open to Research submissions.

We expect that gaining a better understanding of fundamental epigenetic and chromatin processes will result in practical applications in medicine, agriculture and technology. The hope that stem cell research might lead to tissue engineering must be tempered by the realization that iPS cells are genetically identical to the cells that they are derived from and to the subsequent progenitor and differentiated cells that follow, while we know little about the epigenetic processes involved in these transitions. Likewise, the hope that cancer can be treated with epigenetic drugs must be tempered by our very incomplete knowledge of the action of the chromatin targets of these drugs and our realization that they are likely to be only a part of a combination of therapies. While there is great expectation and an understandable emphasis on translational research in many quarters, we believe that the challenges in understanding fundamental epigenetics and chromatin processes remain bottlenecks in achieving these goals. We expect that the free exchange of new results and ideas at this meeting will help us meet these challenges.

## References

[B1] Epigenetics & Chromatin: Interactions and Processeshttp://www.epigeneticsandchromatin.com/conf 10.1186/1756-8935-6-2 PMC3586347 23442888

